# Administration of Human-Derived Mesenchymal Stem Cells Activates Locally Stimulated Endogenous Neural Progenitors and Reduces Neurological Dysfunction in Mice after Ischemic Stroke

**DOI:** 10.3390/cells13110939

**Published:** 2024-05-29

**Authors:** Shuichi Fujiwara, Akiko Nakano-Doi, Toshinori Sawano, Shuji Kubo, Nobutaka Doe, Takayuki Nakagomi

**Affiliations:** 1 Institute for Advanced Medical Sciences, Hyogo Medical University (Nishinomiya Campus), 1-1 Mukogawacho, Nishinomiya 663-8501, Japan; ds22037@hyo-med.ac.jp (S.F.); nakano@hyo-med.ac.jp (A.N.-D.); s-kubo@hyo-med.ac.jp (S.K.); 2 Department of Therapeutic Progress in Brain Diseases, Hyogo Medical University, 1-1 Mukogawacho, Nishinomiya 663-8501, Japan; 3 Department of Biomedical Sciences, Ritsumeikan University, 1-1-1 Nojihigashi, Kusatsu 525-8577, Japan; t-sawano@fc.ritsumei.ac.jp; 4 Department of Rehabilitation, Hyogo Medical University (Kobe Campus), 1-3-6 Minatojima, Chuo-ku, Kobe 650-8530, Japan; doe@hyo-med.ac.jp

**Keywords:** mesenchymal stem cell, neural stem cell, ischemic stroke, transplantation, neurological function

## Abstract

Increasing evidence shows that the administration of mesenchymal stem cells (MSCs) is a promising option for various brain diseases, including ischemic stroke. Studies have demonstrated that MSC transplantation after ischemic stroke provides beneficial effects, such as neural regeneration, partially by activating endogenous neural stem/progenitor cells (NSPCs) in conventional neurogenic zones, such as the subventricular and subgranular zones. However, whether MSC transplantation regulates the fate of injury-induced NSPCs (iNSPCs) regionally activated at injured regions after ischemic stroke remains unclear. Therefore, mice were subjected to ischemic stroke, and mCherry-labeled human MSCs (h-MSCs) were transplanted around the injured sites of nestin–GFP transgenic mice. Immunohistochemistry of brain sections revealed that many GFP^+^ cells were observed around the grafted sites rather than in the regions in the subventricular zone, suggesting that transplanted mCherry^+^ h-MSCs stimulated GFP^+^ locally activated endogenous iNSPCs. In support of these findings, coculture studies have shown that h-MSCs promoted the proliferation and neural differentiation of iNSPCs extracted from ischemic areas. Furthermore, pathway analysis and gene ontology analysis using microarray data showed that the expression patterns of various genes related to self-renewal, neural differentiation, and synapse formation were changed in iNSPCs cocultured with h-MSCs. We also transplanted h-MSCs (5.0 × 10^4^ cells/µL) transcranially into post-stroke mouse brains 6 weeks after middle cerebral artery occlusion. Compared with phosphate-buffered saline-injected controls, h-MSC transplantation displayed significantly improved neurological functions. These results suggest that h-MSC transplantation improves neurological function after ischemic stroke in part by regulating the fate of iNSPCs.

## 1. Introduction

Ischemic stroke is a disease that frequently accompanies neurological deficits, such as sequelae. Recent studies have shown that reperfusion therapies, such as recombinant tissue plasminogen activators and neuroendovascular therapies, can rescue patients with stroke without accompanying severe neurological sequelae [1,2,3]. However, because of the limited time window for reperfusion therapy after stroke onset, it is speculated that a large population of patients with stroke do not receive these therapies. Thus, the development of alternative therapies, such as stem cell-based therapies, which have a broader therapeutic window, is required.

Mesenchymal stem cells (MSCs) are multipotent stem cells that can repair damaged tissues following injury. Accumulating evidence shows that MSC transplantation is a promising therapy for ischemic stroke [4]. Studies have shown that transplanted MSCs transdifferentiated into neural cells with neurological functional improvement [5]. Furthermore, MSC transplantation following ischemic stroke promoted reparative processes through immunomodulatory effects [6,7] and angiogenesis [8]. Moreover, MSC-derived factors (e.g., neurotrophic factors [9], extracellular vesicle [7,10], and microRNA [11]) are involved in neural regeneration with functional improvement. Thus, it is speculated that transplanted MSCs have a positive effect on damaged brains after stroke through multiple mechanisms. However, the mechanism by which MSCs exert a positive effect on neural repair and neurological functional improvement after ischemic stroke remains unclear.

Following brain injuries such as ischemic stroke, endogenous neural stem/progenitor cells (NSPCs) are activated in neurogenic zones, such as the subventricular zone (SVZ) [12]. Studies have shown that transplanted MSCs promoted neurogenesis by activating NSPCs in the SVZ and subgranular zones (SGZ) [13,14,15]. However, increasing evidence shows that the migratory capacity of SVZ-derived NSPCs is limited [16] and that locally activated NSPCs, rather than SVZ-derived NSPCs, are involved in neural repair after ischemic stroke [17]. Although the precise roles of locally activated NSPCs remain unclear, we previously demonstrated that injury/ischemia-induced NSPCs (iNSPCs) can differentiate into electrophysiologically functional neurons [18] and that they contribute to neural repair after brain injuries [19]. Thus, endogenous iNSPC augmentation would be useful for promoting neural regeneration after an ischemic stroke.

In this study, using bone marrow-derived human MSCs (h-MSCs), we transplanted mCherry-labeled h-MSCs into nestin–GFP transgenic (TG) mice after ischemic stroke and investigated whether MSC transplantation activates endogenous iNSPCs. We found that MSC transplantation increased the number of GFP^+^ iNSPCs around the transplanted sites with neurological functional improvement. Furthermore, coculture studies have shown that h-MSCs promoted the proliferation and neurite growth of iNSPCs and regulated gene expressions relevant to self-renewal, neural differentiation, and synapse formation. These results indicate that MSC transplantation has a positive effect on neural regeneration, in part, by regulating the fate of iNSPCs.

## 2. Materials and Methods

### 2.1. Animal Studies

In this study, 6–10-week-old adult mice [C57BL/6JJcl mice (Clea Japan Inc., Tokyo, Japan); CB-17/Icr-+/+Jcl mice (Clea Japan Inc.); CB-17/Icr-scid/scid Jcl mice (Clea Japan Inc.); and B6.Cg-Tg(Nes-EGFP)1Yamm mice, which were produced in association with the National Bioresource Project of the MEXT/AMED, Japan [20] (RIKEN BioResource Research Center, Ibaraki, Japan)], were used for all experiments.

In some experiments, B6.Cg-Tg(Nes-EGFP)1Yamm mice [nestin–GFP TG mice (C57BL/6 background)] were crossed with CB-17/Icr-+/+Jcl mice (CB-17 wild-type mice) using backcrossing techniques, as previously described [17,21]. In this study, we established nestin–GFP TG mice (CB-17 background) that were highly reproducible [17] and used this background of mice to trace the fate of nestin^+^ iNSPCs following h-MSC transplantation.

### 2.2. Induction of Ischemic Stroke

Experimental procedures were approved by the Animal Care Committee of Hyogo Medical University (approval numbers: 16-059; 18-061; 18-074; 2019-10-3; 22-019AG). Permanent focal cerebral ischemia was induced in CB-17/Icr-scid/scid Jcl mice or nestin–GFP TG mice (CB-17 background) by occlusion of the middle cerebral artery (MCA). In brief, the mice were subjected to middle cerebral artery occlusion (MCAO) under isoflurane anesthesia by ligating and interrupting the distal portion of the left MCA, as previously described [17,19,21,22]. Immediately following MCAO, all mice received antibiotics. This strain of mice showed high survival rates, as described previously [17,19].

### 2.3. Cell Transplantation

Human bone marrow-derived mesenchymal stem cells (h-MSCs; PT-2501, Lonza, Basil, Switzerland) were maintained in media according to the manufacturer’s instructions and used in this study. Fluorescence-activated cell sorting (FACS) analysis confirmed that h-MSCs expressed various MSC markers, including CD44, CD90, CD105, and CD166, which is consistent with previous findings [23,24]. We transplanted h-MSCs or h-MSCs transfected with mCherry-expressing lentivirus vectors (mCherry^+^ h-MSCs) into CB-17/Icr-scid/scid Jcl mice (immunodeficient mice) or nestin–GFP TG mice, as previously described [21]. Briefly, under isoflurane anesthesia, h-MSCs (0.5 μL; 1.0 × 10^5^ cells/μL) or mCherry^+^ h-MSCs (0.5 μL; 1.0 × 10^5^ cells/μL) were transplanted transcranially into peri-ischemic areas (coordinates from bregma: anteroposterior, 0 mm; mediolateral, +2.5 mm; dorsoventral, −2.5 mm) 6 weeks after MCAO. Control mice received 0.5 μL of phosphate-buffered saline (PBS) 6 weeks after MCAO, as previously described [21].

### 2.4. Behavioral Tests

Neurological function was evaluated using CB-17/Icr-scid/scid Jcl mice divided into three categories [sham-operated mice (sham group), PBS-injected mice after MCAO (PBS group), and h-MSC-administered mice after MCAO (h-MSC group)] 2–9 weeks after treatment (8–15 weeks post-MCAO or sham surgery). Behavioral tests were performed, as previously described [19,21].

Briefly, spontaneous locomotive behavior was evaluated using a cubic (30 × 30 × 30 cm) open-field apparatus made of transparent acrylic plates (Taiyo Electric Co., Ltd., Osaka, Japan). The infrared beams were attached on the lateral side of the open field box, and the circuit was constructed between the two beams. Mice were placed into the open field box and allowed to move freely for 10 min per day for two consecutive days. Crossing the circuit was counted as locomotor activity, and the total number of crossings was determined. 

Spatial working memory was assessed using a Y-shaped maze with three identical arms (length: 40 cm; width: 3 cm; height: 20 cm). The arms were labeled A, B, and C, each diverging at 120° angles from a central area. Mice were placed in the central area and allowed to move freely for 5 min. The behaviors of the mice were recorded with a digital video camera placed above the maze. When all four paws of a mouse touched down an arm runway, the activity was regarded as having entered the arm. An alternation was defined when mice entered into all three different arms consecutively (e.g., ABC, CBA, BAC). The percentage of alternation (the alternation rate) was calculated using the following equation: alternation rate = (number of alternations/the total number of arm entries − 2) × 100.

To assess long-term memory, mice were trained in the passive avoidance apparatus composed of light and dark compartments (15 × 15 × 15 cm each) separated by a door. In day conditioning, mice were placed in the light compartment for 10 s, after which the door was opened. If mice were moved into the dark compartment, the door was closed, and an electrical shock (140 V, 5 s) was administered. In the test trial, the mice were placed in the light compartment 24 h later. Then, the latency to enter the dark compartment was assessed with a maximum limit of 3 min.

The forced swimming test was conducted to evaluate depression-like states. A small cylindrical tank (inside diameter: 18 cm; depth: 29 cm) was filled with water up to 15 cm deep and maintained at 24 °C ± 1 °C. Then, mice were placed into the tank. The duration of immobility was measured for 6 min automatically using an activity monitoring system equipped with an infrared detector (SUPERMEX, CompACT FSS ver. 2, Muromachi Kikai Co., Tokyo, Japan).

### 2.5. Preparation of Brain Samples Following Ischemic Stroke

The mice were anesthetized intraperitoneally with a mixture that included medetomidine, midazolam, and butorphanol, followed by transcardial perfusion with 4% paraformaldehyde (PFA), as previously described [17,21,22]. The brains were then removed and fixed with 4% PFA. The fixed brains were cryoprotected in 30% sucrose, frozen at −80 °C, and cut into 20 μm coronal sections using a cryostat, followed by immunohistochemical staining.

### 2.6. Immunohistochemistry

Immunohistochemistry was performed as described previously [17,19,21,22]. In brief, coronal brain sections (20 μm thick) were stained with primary antibodies against GFP [1:2000, chicken, Abcam (ab13970), Cambridge, UK] and mCherry [1:1000, rabbit, Abcam (ab167453)], followed by Alexa Fluor 488- or 555-conjugated secondary antibodies (1:500, Molecular Probes, Eugene, OR, USA). Nuclei were counterstained with 4′,6-diamidino-2-phenylindole (DAPI; 1:500, Kirkegaard & Perry Laboratories, Inc., Gaithersburg, MD, USA). Images were captured using a confocal laser microscope (LSM780; Carl Zeiss AG, Oberkochen, Germany).

### 2.7. Cell Culture

Endogenous iNSPCs were extracted from ischemic areas of the brain cortices of post-stroke mice and maintained in Dulbecco’s Modified Eagle’s medium/F12 (DMEM/F12; Thermo Fisher Scientific, Waltham, MA, USA), which consisted of basic fibroblast growth factor (bFGF; 20 ng/mL; Peprotech, Rocky Hill, NJ, USA), epidermal growth factor (EGF; 20 ng/mL; Peprotech), N_2_ (1%; Thermo Fisher Scientific), and 2% fetal bovine serum (FBS), as previously described [18,21,22]. iNSPCs were used for the experiments using passages 9–11.

To investigate the effects of h-MSCs on iNSPC proliferation, iNSPCs were cocultured with h-MSCs transfected with GFP-expressing lentivirus (GFP^+^ h-MSCs), as previously described [21,22]. In brief, iNSPCs (1.0 × 10^4^ cells/well) were plated on poly-D-lysine-coated 6-well dishes in DMEM/F12 medium containing bFGF, EGF, N_2_, and 2% FBS. One hour later, GFP^+^ h-MSCs (1.0 × 10^4^ cells/well) were plated onto the same dishes. On day 7 after incubation, the cells were fixed and stained with antibodies against GFP (1:2000, chicken, Abcam), nestin (1:200, mouse, Millipore, Billerica, MA, USA), Sox2 (1:100, rabbit, Abcam), and Ki67 (1:100, mouse, BD Pharmingen, San Diego, CA, USA), followed by incubation with Alexa Fluor 488- or 555-conjugated secondary antibodies (1:500, Molecular Probes), as previously described [21,22]. Then, the number of nestin^+^ iNSPCs (GFP^−^/nestin^+^ cells) or Sox2^+^ iNSPCs (GFP^−^/Sox2^+^ cells) was evaluated between iNSPC monoculture (controls) and iNSPCs cocultured with GFP^+^ h-MSCs using 12 data points [4 areas/sample, 3 samples/each marker (*n* = 3)]. Furthermore, the ratio of Ki67^+^ iNSPCs (GFP^−^/Ki67^+^ cells to GFP^−^/DAPI^+^ cells) was evaluated between iNSPC monocultures and cocultures with GFP^+^ h-MSCs using 12 data points [4 areas/sample, 3 samples/each marker (*n* = 3)].

To examine the effects of h-MSCs on endogenous iNSPC differentiation, neurospheres derived from iNSPCs were cultured alone or cocultured with h-MSCs, as previously described [21,22]. Briefly, h-MSCs (1 × 10^4^ cells/well) were plated into poly-L-lysine-coated 24-well dishes with medium to maintain MSCs. One day later, the medium was replaced with a neurobasal medium (Thermo Fisher Scientific) with bFGF, B-27 supplement (Thermo Fisher Scientific), and 2% FBS. Neurospheres (approximately 5–10/well) were added to the dishes. As a control, neurospheres (approximately 5–10/well) alone were plated into poly-L-lysine-coated 24-well dishes in a neurobasal medium with bFGF, B-27 supplement, and 2% FBS. On day 9 after seeding the neurospheres, the cells were fixed and stained with antibodies against GFP (1:2000, chicken, Abcam), STEM 121 (1:500, mouse, Takara Bio Inc., Shiga, Japan), Tuj1 (1:2000, rabbit, Abcam), and GFAP (1:500, rabbit, Abcam), followed by incubation with Alexa Fluor 488- or 555-conjugated secondary antibodies (1:500, Molecular Probes). The areas of GFP^+^ neurites were evaluated for total neurospheres per group obtained from 3 wells/each group (*n* = 18 for iNSPCs alone (controls); *n* = 25 for iNSPCs co-incubated with h-MSCs) using ImageJ, as previously described [22]. Furthermore, the ratio of iNSPC-derived neurons (GFP^−^/Tuj1^+^ cells to GFP^−^/DAPI^+^ cells) or iNSPC-derived astrocytes (GFP^−^/GFAP^+^ cells to GFP^−^/DAPI^+^ cells) was evaluated between controls (iNSPC-derived neurospheres alone) and cocultures with GFP^+^ h-MSCs using 9 data points (3 areas/sample, 3 samples/each marker [*n* = 3]).

### 2.8. Microarray Analysis

iNSPCs monocultures or cocultures with GFP^+^ h-MSCs were subjected to FACS. iNSPCs (2.0 × 10^4^ cells/well) were plated into poly-D-lysine-coated 6-well dishes in DMEM/F12 medium containing bFGF, EGF, N_2_, and 2% FBS. After 1 h, GFP^+^ h-MSCs (2.0 × 10^4^ cells/well) were plated into the same dishes and incubated for 7 days. After removing GFP^+^ cells using FACS procedures, iNSPCs were selectively collected (2.1 × 10^5^ cells/well in monocultures; 9.6 × 10^5^ cells/well in cocultures).

Total RNA was then isolated from iNSPCs using the RNeasy Micro Kit (Qiagen, Hilden, Germany), as previously described [17,22]. RNA samples (*n* = 1, for each group) were subjected to microarray analysis, and the results were analyzed using the Affymetrix transcriptome analysis console, as previously described [17,21,22]. Pathway analysis was performed using WikiPathways, as previously described [25]. GO analysis was performed using the “GO enrichment analysis and visualization tool (Gorilla)”, as previously described [26].

### 2.9. Statistical Analysis

Data are presented as means ± standard errors of the mean. Differences between two groups or samples were evaluated using Student’s *t*-test. Behavioral tests were analyzed among the sham, PBS, and h-MSC groups using one-way analysis of variance (ANOVA), followed by multiple comparisons tests, or repeated-measures ANOVA, including factors, as previously described [19,21]. *p*-values < 0.05 were used to denote statistical significance.

## 3. Results

### 3.1. h-MSC Transplantation Improves Neurological Function after Ischemic Stroke

**Figure 1:**
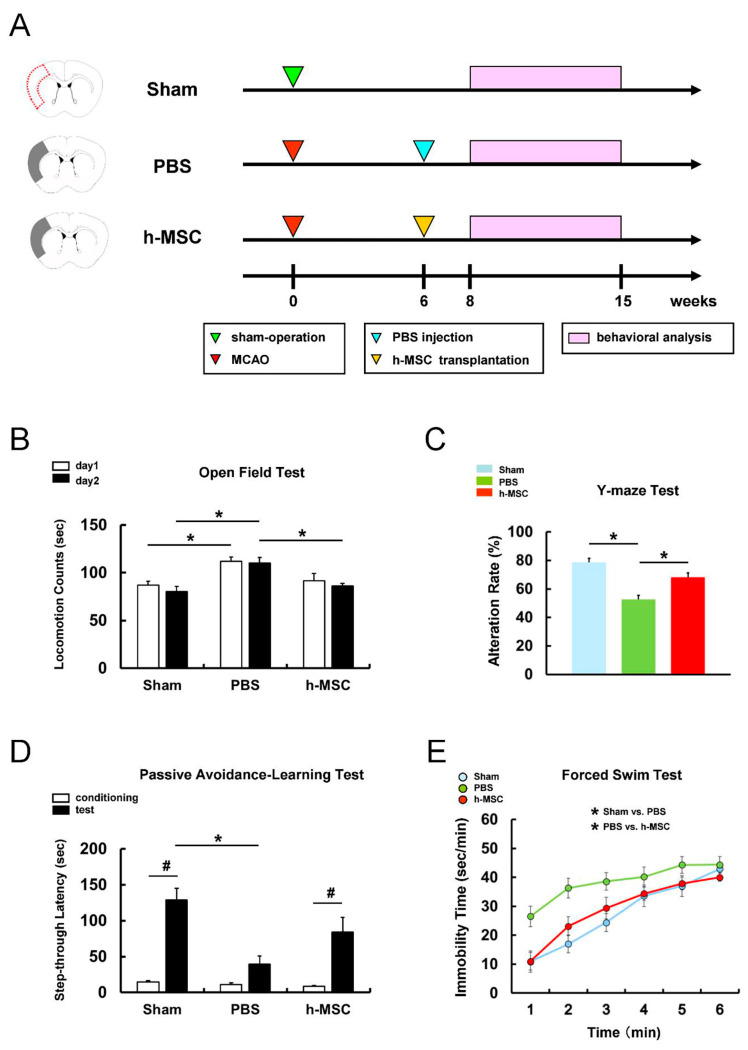
(**A**) Performance metrics on tests were compared among the three mouse treatment groups: (1) Sham surgery group (*n* = 8); (2) MCAO followed by PBS injection (PBS group, *n* = 10); and (3) MCAO followed by h-MSC injection (h-MSC group, *n* = 8). (**B**–**E**) Performance levels on the open-field (**B**), Y-maze (**C**), passive avoidance-learning (**D**), and forced swim tests (**E**) among the three mouse treatment groups. * *p* < 0.05 between groups on the same day (**B**). * *p* < 0.05 between groups (**C**–**E**). ^#^ *p* < 0.05 between the conditioning and test trials within groups (**D**). Abbreviations: MCAO, middle cerebral artery occlusion; MSC, mesenchymal stem cell; PBS, phosphate-buffered saline.

First, to investigate whether h-MSC transplantation can improve neurological function after ischemic stroke, mice were divided into three groups: (1) sham-operation (sham group; *n* = 8), (2) PBS injection after MCAO (PBS group; *n* = 10), and (3) h-MSC administration (5.0 × 10^4^ cells) after MCAO (h-MSC group; *n* = 8) (Figure 1A). Following PBS or h-MSC treatment, neurological function was assessed in mice at 2–9 weeks using a behavioral test battery (Figure 1A), as described previously [19,21].

The open-field test was performed for 2 consecutive days to evaluate spontaneous locomotor activity (Figure 1B). On days 1 and 2, locomotor activity was significantly higher in the PBS group than in the sham group, indicating that stroke-associated hyperactivity was induced in mice after ischemia. However, locomotor activity on day 2 was significantly lower in the h-MSC group than in the PBS group (Figure 1B), indicating that h-MSC transplantation suppressed stroke-associated hyperactivity.

The Y-maze test (Figure 1C) showed that the alternation rate was significantly reduced in the PBS group compared with that in the sham group, indicating that ischemic stroke-induced working memory impairment in mice after MCAO. However, the alternation rate was significantly higher in the h-MSC group than in the PBS group, indicating that h-MSC transplantation improved this memory disturbance (Figure 1C).

Furthermore, long-term associative memory was assessed using the passive avoidance-learning test (Figure 1D). The latency to enter the chamber was significantly longer on the test day than on the conditioning day in the sham group (Figure 1D). In contrast, the latency to enter the chamber was not significantly different between the conditioning and test days in the PBS groups, indicating ischemic stroke-induced memory deficit after MCAO. However, the latency to enter the chamber was significantly longer on the test day than on the conditioning day in the h-MSC group (Figure 1D). These results indicate that h-MSC transplantation improves memory disturbance after ischemic stroke.

The time spent in the immobilized state was assessed using the forced swim test to evaluate the presence of depression-like symptoms following ischemic stroke (Figure 1E). Although the immobility time was significantly longer in the PBS group than in the sham group, the immobility time was significantly shorter in the h-MSC group than in the PBS group (Figure 1E). These results indicate that h-MSC transplantation improves depression-like symptoms associated with ischemic stroke.

### 3.2. h-MSCs Transplanted into Mouse Brains Activate Endogenous iNSPCs in Mice after Ischemic Stroke

**Figure 2:**
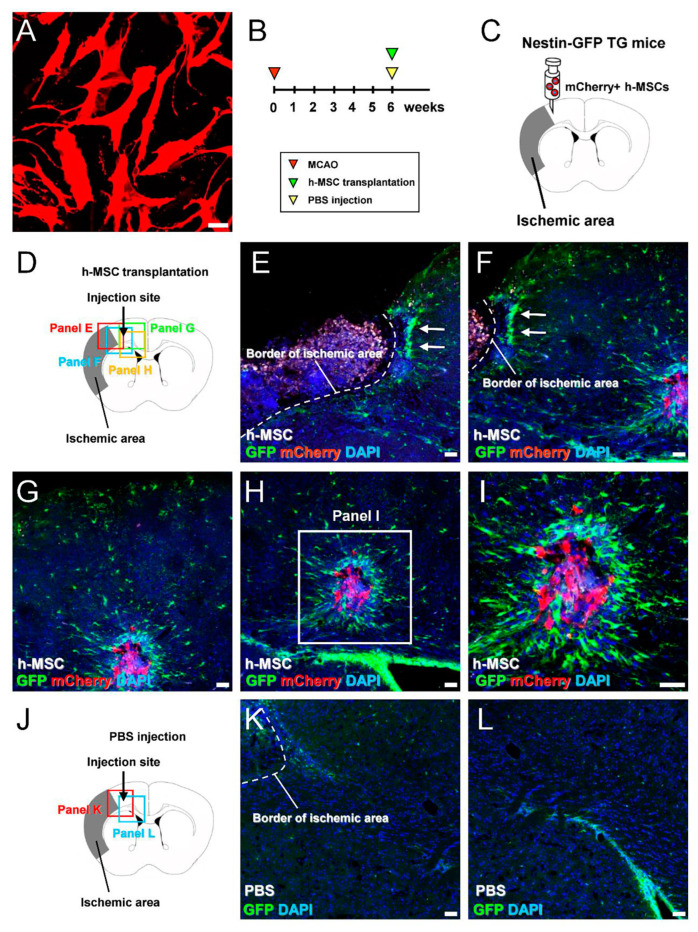
(**A**,**B**) mCherry-labeled h-MSCs (**A**) were grafted 6 weeks after MCAO induction (**B**). (**C**) mCherry^+^ h-MSCs were transplanted around ischemic areas of nestin–GFP transgenic mice. (**D**–**I**) Immunohistochemical analysis performed 3 days after transplantation revealed that GFP^+^ iNSPCs were located at peri-ischemic areas (**E**,**F**: arrows) and around grafted mCherry^+^ h-MSCs (**F**–**I**) [GFP (**E**–**I**: green); mCherry (**E**–**I**: red); DAPI (**E**–**I**: blue)]. (**J**–**L**) Immunohistochemistry 3 days after PBS injection revealed that few GFP^+^ cells were observed at the injection site (**K**); however, many GFP^+^ cells were observed in the SVZ (**L**) [GFP (**K**,**L**: green); DAPI (**K**,**L**: blue)]. Scale bars: 50 µm (**A**,**E**–**I**,**K**,**L**). Abbreviations: DAPI, 4′,6-diamidino-2-phenylindole; GFP, green fluorescent protein; MSC, mesenchymal stem cell; MCAO, middle cerebral artery occlusion; iNSPC, injury/ischemia-induced neural stem/progenitor cell; PBS, phosphate-buffered saline; SVZ, subventricular zone.

Thus far, our data showed that MSC transplantation improved neurological dysfunction after ischemic stroke. To elucidate the underlying mechanism, we investigated the effects of MSC transplantation on endogenous iNSPCs. h-MSCs were transplanted into nestin–GFP TG mice whose ischemic areas were highly reproducible [17]. Six weeks after MCAO, mCherry-labeled h-MSCs (mCherry^+^ h-MSCs; 5.0 × 10^4^ cells/µL) (Figure 2A) were transplanted into nestin–GFP TG mice (Figure 2B,C). On day 3, after h-MSC or PBS treatment, the brains were removed, and brain sections were subjected to immunohistochemistry (Figure 2D–L).

Using the nestin–GFP TG mice [17], we previously showed that GFP^+^ iNSPCs were activated within and around the ischemic areas during the acute phase. However, GFP^+^ iNSPCs gradually decreased and localized around the ischemic areas at later time points. Consistent with these findings, immunohistochemical analysis performed during the chronic period revealed that GFP^+^ cells were located at peri-ischemic areas (Figure 2E). Apart from the GFP^+^ cells at peri-ischemic areas, many GFP^+^ cells were observed near mCherry^+^ h-MSCs that were transplanted around the ischemic areas (Figure 2H,I). In addition, GFP^+^ cells in the SVZ likely did not migrate into the injected sites and were instead located in situ (Figure 2H), similar to those observed in PBS-injected controls (Figure 2J–L). These results indicate that h-MSC transplantation reactivates regionally stimulated endogenous iNSPCs.

### 3.3. h-MSCs Promote the Proliferation of iNSPCs

**Figure 3:**
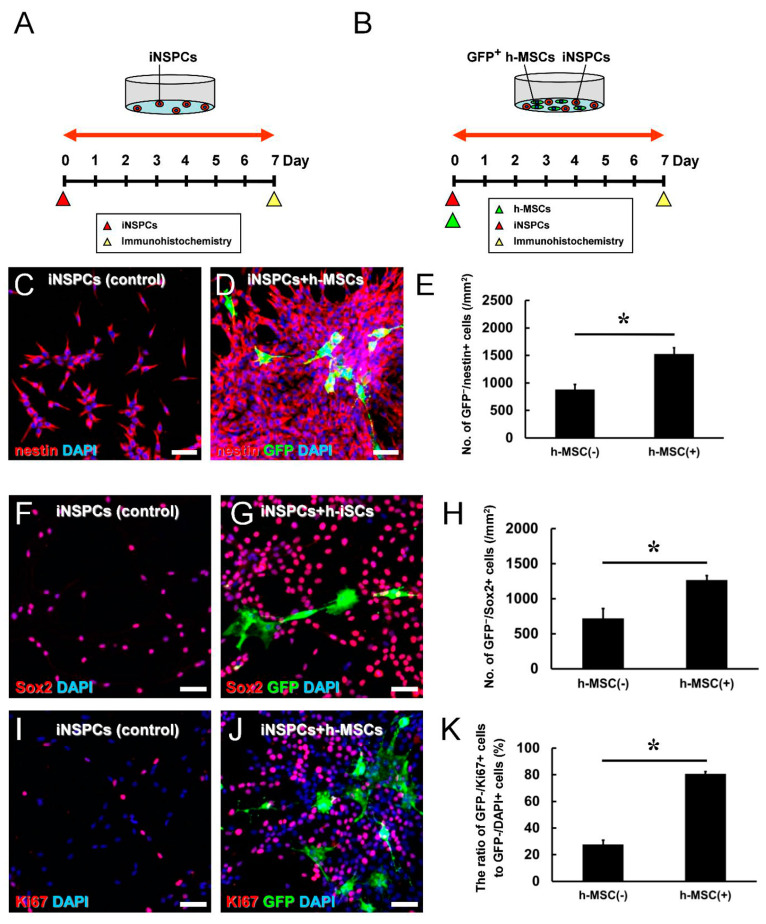
(**A**,**B**) Assessment of proliferative activities of iNSPCs in monoculture (**A**) or coculture with h-MSCs (**B**). (**C**–**E**) Immunocytochemistry revealed that the number of iNSPC-derived nestin^+^ cells (GFP^−^/nestin^+^ cells) was significantly higher in coculture with GFP^+^ h-MSCs [h-MSC(+) group] than in iNSPC monoculture (control) [h-MSC(−) group] [nestin (**C**,**D**: red); GFP (**D**: green); DAPI (**C**,**D**: blue)]. (**F**–**H**) Immunocytochemistry revealed that the number of iNSPC-derived Sox2^+^ cells (GFP^−^/Sox2^+^ cells) was significantly higher in coculture with GFP^+^ h-MSCs than in iNSPC monoculture [Sox2 (**F**,**G**: red); GFP (**G**: green); DAPI (**F**,**G**: blue)]. (**I**–**K**) Immunocytochemistry revealed that the ratio of iNSPC-derived proliferative cells (GFP^−^/Ki67^+^ cells to GFP^−^/DAPI^+^ cells) was significantly higher in coculture with GFP^+^ h-MSCs than in iNSPC monoculture [Ki67 (**I**,**J**: red); GFP (**J**: green); DAPI (**I**,**J**: blue)]. Scale bars: 50 µm (**C**,**D**,**F**,**G**,**I**,**J**). * *p* < 0.05 between the h-MSC(−) and h-MSC(+) groups (**E**,**H**,**K**). *n* = 3 (12 data points) for each group (**E**,**H**,**K**). Abbreviations: DAPI, 4′,6-diamidino-2-phenylindole; GFP, green fluorescent protein; MSC, mesenchymal stem cell; iNSPC, injury/ischemia-induced neural stem/progenitor cell.

To investigate whether h-MSCs activate endogenous iNSPCs, mouse-brain-derived iNSPCs were isolated from ischemic areas and incubated with h-MSCs under direct cell–cell contact. In brief, iNSPCs were plated on the dishes, followed by further incubation with GFP^+^ h-MSCs 1 h later. iNSPC monocultures (Figure 3A) and cocultures with GFP^+^ h-MSCs (Figure 3B) were incubated for 7 days. On day 7, after incubation, the cells were fixed and subjected to immunohistochemistry. Virtually all iNSPCs (GFP^−^ cells) expressed nestin (Figure 3C,D) and Sox2 (Figure 3F,G). In addition, compared with iNSPCs alone (controls) (Figure 3C,E), a significantly higher number of nestin^+^ iNSPCs (GFP^−^/nestin^+^ cells) were observed in iNSPCs cocultured with h-MSCs (Figure 3D,E). Similar to these findings, compared with iNSPCs alone (Figure 3F,H), a significantly higher number of Sox2^+^ iNSPCs (GFP^−^/Sox2^+^ cells) were observed in iNSPCs after h-MSC treatment (Figure 3G,H). Furthermore, compared with iNSPCs alone (Figure 3I,K), a significantly higher ratio of Ki67^+^ iNSPCs (GFP^−^/Ki67^+^ cells to GFP^−^/DAPI^+^ cells) was observed in iNSPCs cocultured with h-MSCs (Figure 3J,K). These results show that h-MSCs can increase the number of iNSPCs, in part, by promoting their proliferation.

### 3.4. h-MSCs Promote the Differentiation of iNSPCs

**Figure 4:**
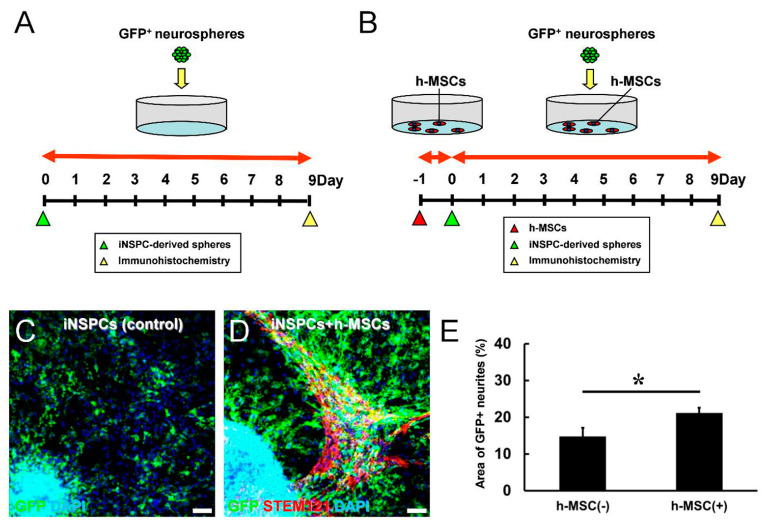
(**A**,**B**) Evaluation of the differentiation potential of GFP^+^ iNSPC-derived neurospheres in monoculture (**A**) and in coculture with h-MSCs (**B**). (**C**–**E**) Immunocytochemistry revealed that the ratio of GFP^+^ iNSPC-derived differentiated cells (GFP^+^ area to total area) was significantly higher in coculture with STEM121^+^ h-MSCs than in GFP^+^ iNSPCs alone (control) [GFP (**C**,**D**: green); STEM121 (**D**: red); DAPI (**C**,**D**: blue)]. Scale bars: 100 µm (**C**,**D**). * *p* < 0.05 between the h-MSC(−) and h-MSC(+) groups (**E**). *n* = 18 for h-MSC(−); *n* = 25 for h-MSC(+) groups (**E**). Abbreviations: DAPI, 4′,6-diamidino-2-phenylindole; GFP, green fluorescent protein; iNSPC, injury/ischemia-induced neural stem/progenitor cell; MSC, mesenchymal stem cell.

We next investigated whether h-MSCs promote the differentiation of iNSPCs. Therefore, h-MSCs were plated onto dishes. One day later, GFP^+^ neurospheres derived from iNSPCs were plated onto dishes without h-MSCs (controls) (Figure 4A) or with h-MSCs (Figure 4B). On day 9, after incubation, cells were fixed and subjected to immunohistochemistry using antibodies against GFP and STEM121. The latter can react with human-derived cells; thus, h-MSCs were detectable by this antibody. Immunostaining revealed that although GFP^+^ neurites were observed in controls (Figure 4C) and coculture with h-MSCs (Figure 4D), the areas of GFP^+^ neurites were significantly increased in the presence of h-MSCs (Figure 4E). These results indicate that h-MSCs promote the differentiation of iNSPCs.

**Figure 5:**
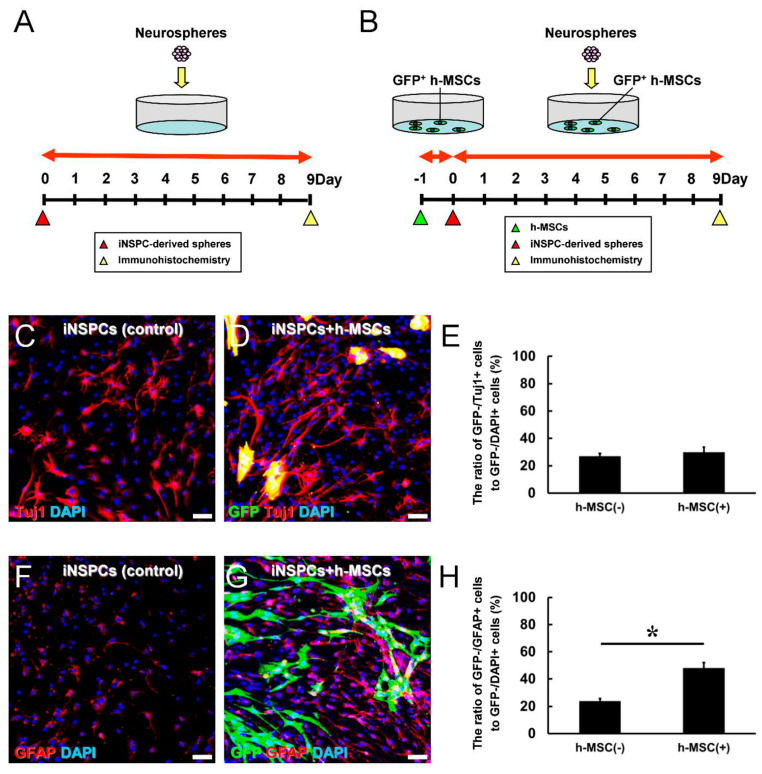
(**A**,**B**) Assessment of the differentiated neural lineages of iNSPC-derived neurospheres in monoculture (**A**) and in coculture with h-MSCs (**B**). (**C**,**D**) Immunocytochemistry revealed that iNSPC-derived neurons (GFP^−^/Tuj1^+^ cells) were observed in monoculture (**C**) and in coculture with h-MSCs (**D**) [Tuj1 (**C**,**D**: red); GFP (**D**: green); DAPI (**C**,**D**: blue)]. (**E**) The ratio of iNSPC-derived neurons (GFP^−^/Tuj1^+^ cells to GFP^−^/DAPI^+^ cells) did not differ significantly between the control and coculture with h-MSCs. (**F**,**G**) Immunocytochemistry revealed that iNSPC-derived astrocytes (GFP^−^/GFAP^+^ cells) were observed in monoculture (**F**) and in coculture with h-MSCs (**G**) [GFAP (**F**,**G**: red); GFP (**G**: green); DAPI (**F**,**G**: blue)]. (**H**) The ratio of iNSPC-derived astrocytes (GFP^−^/GFAP^+^ cells to GFP^−^/DAPI^+^ cells) was significantly higher in the coculture with h-MSCs than in the control. Scale bars: 50 µm (**C**,**D**,**F**,**G**). * *p* < 0.05 between the h-MSC(−) and h-MSC(+) groups (**H**). *n* = 3 (9 data points) for each group (**E**,**H**). Abbreviations: DAPI, 4′,6-diamidino-2-phenylindole; GFAP, glial fibrillary acidic protein; GFP, green fluorescent protein; iNSPC, injury/ischemia-induced neural stem/progenitor cell; MSC, mesenchymal stem cell.

Thus, we assessed the neural cell types (e.g., neurons, astrocytes) that were significantly upregulated in the presence of h-MSCs. Therefore, GFP^+^ h-MSCs were plated onto the dishes. One day later, neurospheres derived from iNSPCs were plated onto dishes without GFP^+^ h-MSCs (controls) (Figure 5A) or with GFP^+^ h-MSCs (Figure 5B). On day 9, after incubation, cells were fixed and subjected to immunohistochemistry. Immunostaining revealed that iNSPCs monocultured or cocultured with GFP^+^ h-MSCs expressed the neuronal marker Tuj1 (Figure 5C,D) and the astrocytic marker GFAP (Figure 5F,G). Although the ratio of iNSPC-derived neurons (GFP^−^/Tuj1^+^ cells to GFP^−^/DAPI^+^ cells) did not differ significantly between controls and cocultures with h-MSCs (Figure 5E), the ratio of iNSPC-derived astrocytes (GFP^−^/GFAP^+^ cells to GFP^−^/DAPI^+^ cells) was significantly higher in cocultures with h-MSCs than in controls (Figure 5H). Although the ratio of iNSPC-derived neurons did not differ significantly between the two groups (Figure 5E), the presence of h-MSCs significantly increased the number of neural differentiated cells (Figure 4E). Therefore, h-MSCs may promote neuron generation and astrocyte production.

### 3.5. h-MSCs Regulate Genes Related to Self-Renewal and Maintenance in iNSPCs

**Figure 6:**
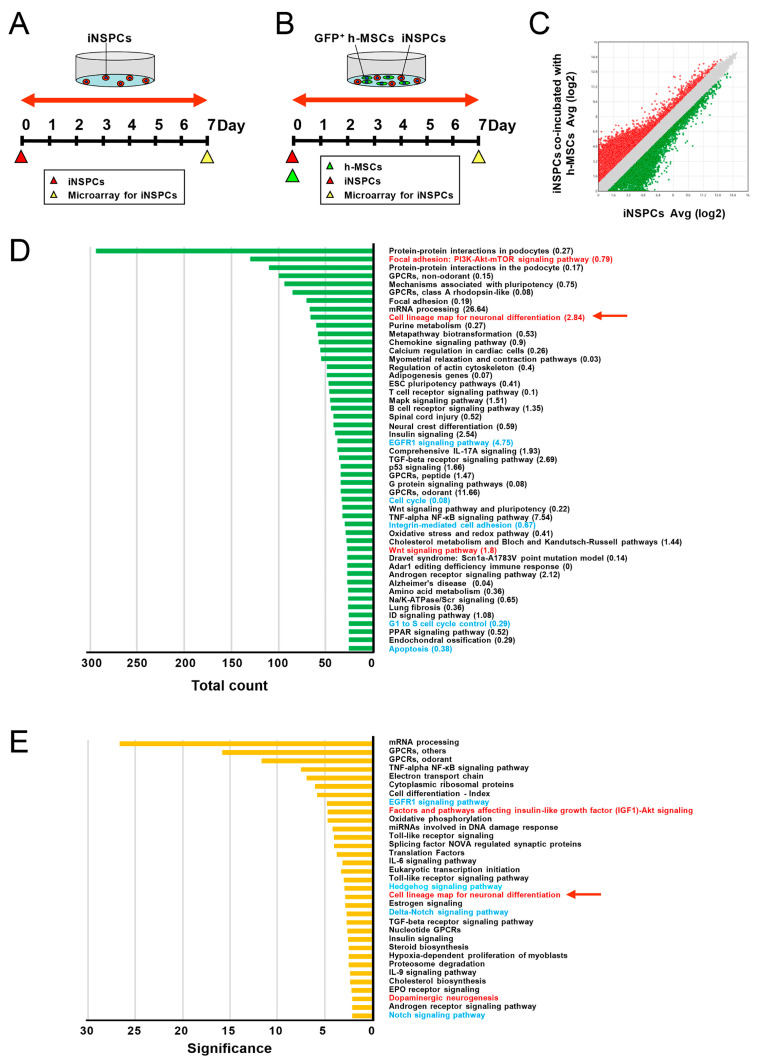
(**A**,**B**) iNSPCs alone (**A**) or cocultures with GFP^+^ h-MSCs (**B**) were subjected to microarray analysis after removing GFP^+^ cells by FACS. (**C**) Scatter plot analysis revealed the distribution of genes > 2-fold higher (red plots) or 2-fold lower (green plots) in iNSPCs after coincubation with h-MSCs than in iNSPCs alone. (**D**,**E**) The target genes (**C**) were subjected to pathway analysis, and the categories were displayed in the order of total “count” up to ≥25 (**D**) or in order of “significance” up to >2-fold (**E**). The term “cell lineage map for neuronal differentiation” was commonly listed (**D**,**E**: red arrows). Abbreviations: FACS, fluorescence-activated cell sorting; GFP, green fluorescent protein; iNSPC, injury/ischemia-induced neural stem/progenitor cell; MSC, mesenchymal stem cell.

Thus far, our data showed that h-MSCs promoted the proliferation and neural differentiation of iNSPCs. To investigate this mechanism, we evaluated changes in the gene expression patterns of iNSPCs after cell–cell contact with h-MSCs. Therefore, iNSPCs monocultured (Figure 6A) or cocultured with h-MSCs (Figure 6B) were sorted by FACS and subjected to microarray analysis.

By targeting all genes that were significantly upregulated (>2-fold, red dots) or downregulated (<−2-fold, green dots) in iNSPCs cocultured with h-MSCs compared with iNSPCs alone (Figure 6C), we investigated the pathways that were significantly different in iNSPCs after cocultures with h-MSCs. Several signaling pathways, such as cell cycle [27], apoptosis [28], growth factor [29], integrin [30,31], Notch-1 [29,32], and Hedgehog [33], are involved in regulating the self-renewal and maintenance of NSPCs. Therefore, to uncover the mechanism by which h-MSCs increase the number of iNSPCs, we examined the presence of these factors.

Pathway analysis was performed, and the categories were shown in order of total “count” (≥25), indicating the number of hit genes in each category (Figure 6D). The results show that several categories that can affect the maintenance of iNSPCs (e.g., “EGFR1 signaling pathway”, “cell cycle”, “integrin-mediated cell adhesion”, “G1 to S cell cycle control”, and “apoptosis”) were included in the list (Figure 6D, labeled by blue font). However, the values of “significance” of these categories displayed in parentheses were <1-fold other than the “EGFR1 signaling pathway”. Thus, the categories by pathway analysis were next shown in order of “significance” up to >2-fold. The results revealed that several categories that can affect the cell numbers of iNSPCs (e.g., the “EGFR1 signaling pathway”, “Hedgehog signaling pathway”, “Delta-Notch signaling pathway”, and “Notch signaling pathway”) were included in the list (Figure 6E, labeled by blue font). These results suggest that h-MSCs partially affect the number of iNSPCs via these signaling pathways.

### 3.6. h-MSCs Regulate Genes Related to Neural Lineage Differentiation and Synapses in iNSPCs

Studies have shown that several signaling pathways, such as the Wnt [34] and Akt signaling pathways [35,36], are involved in the regulation of neural differentiation of NSPCs. Therefore, to investigate the mechanism by which h-MSCs promote the neural differentiation of iNSPCs, we examined the presence of these factors.

The pathway analysis in order of “count” included categories related to neural differentiation (e.g., “factors and pathways affecting insulin-like growth factor (IGF1)–Akt signaling”, “cell lineage map for neuronal differentiation”, and “Wnt signaling pathway”) (Figure 6D, labeled by red font). Furthermore, the pathway analysis in order of “significance” included categories related to neural differentiation (e.g., “factors and pathways affecting IGF1–Akt signaling”, “cell lineage map for neuronal differentiation”, and “dopaminergic neurogenesis”) (Figure 6E, labeled by red font). Because “cell lineage map for neuronal differentiation” was commonly listed, regardless of the order of “count” and “significance” (Figure 6D,E, a red arrow), we investigated the genes in this category in more detail.

**Figure 7:**
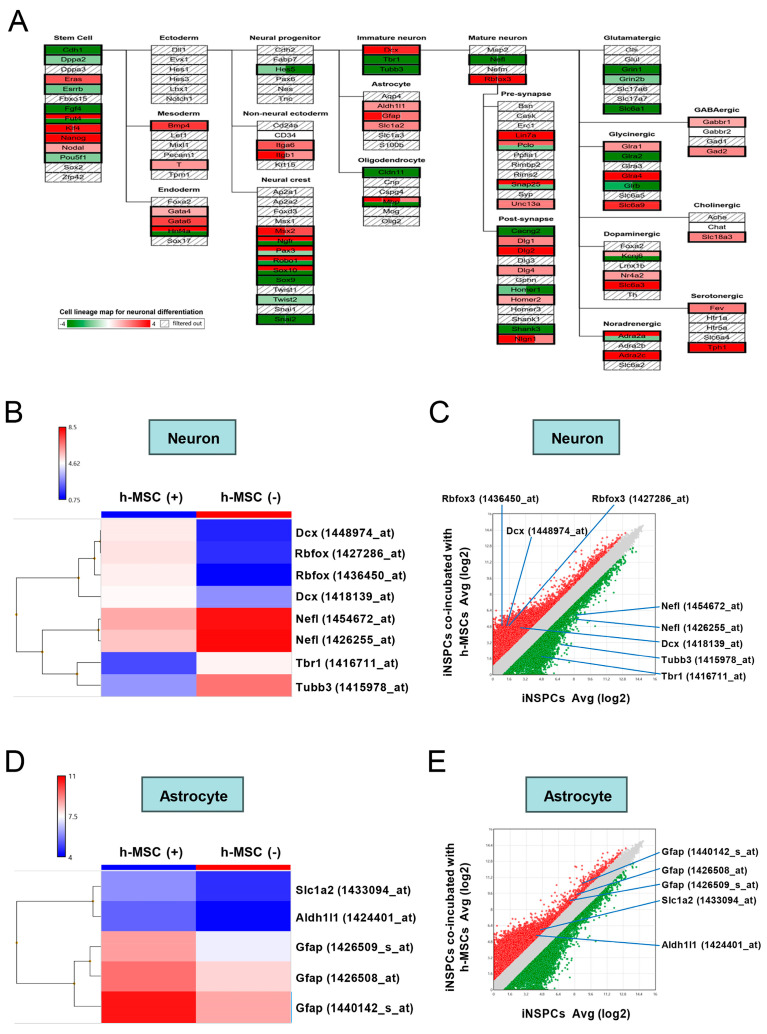
(**A**) Pathway analysis for “cell lineage map for neuronal differentiation” showed that various neural lineage-related genes were upregulated and/or downregulated in iNSPCs after coincubation with h-MSCs relative to those in iNSPCs alone. (**B**,**D**) Heat map analysis of the gene expression patterns for neurons (**B**) and astrocytes (**D**) in iNSPCs alone and coincubation with h-MSCs. (**C**,**E**) Scatter plot analysis reveals that the genes associated with neurons (**C**) or astrocytes (**E**) are located at the regions >2-fold higher (red plots) or 2-fold lower (green plots) in iNSPCs after coincubation with h-MSCs relative to iNSPCs alone. Abbreviations: iNSPC, injury/ischemia-induced neural stem/progenitor cell; MSC, mesenchymal stem cell.

The pathway (Figure 7A), heatmapping (Figure 7B), and scatter plot analysis (Figure 7C) showed that various neuron-associated genes (e.g., *Dcx*, *Tbr1*, *Tubb3*, *Nefl*, and *Rbfox3*) were significantly upregulated (>2-fold, red dots in Figure 7C) and/or downregulated (<−2-fold, green dots in Figure 7C) in cocultures with h-MSCs compared with those in controls. Furthermore, the pathway (Figure 7A), heatmapping (Figure 7D), and scatter plot analysis (Figure 7E) showed that several astrocytic markers, such as *GFAP*, *Aldh1l1*, and *Slc1a2*, were significantly upregulated (>2-fold, red dots in Figure 7E) in cocultures with h-MSCs compared with controls. These results indicate that h-MSCs regulate the differentiation of iNSPCs into neural cells, including astrocytic and neuronal lineages.

**Figure 8:**
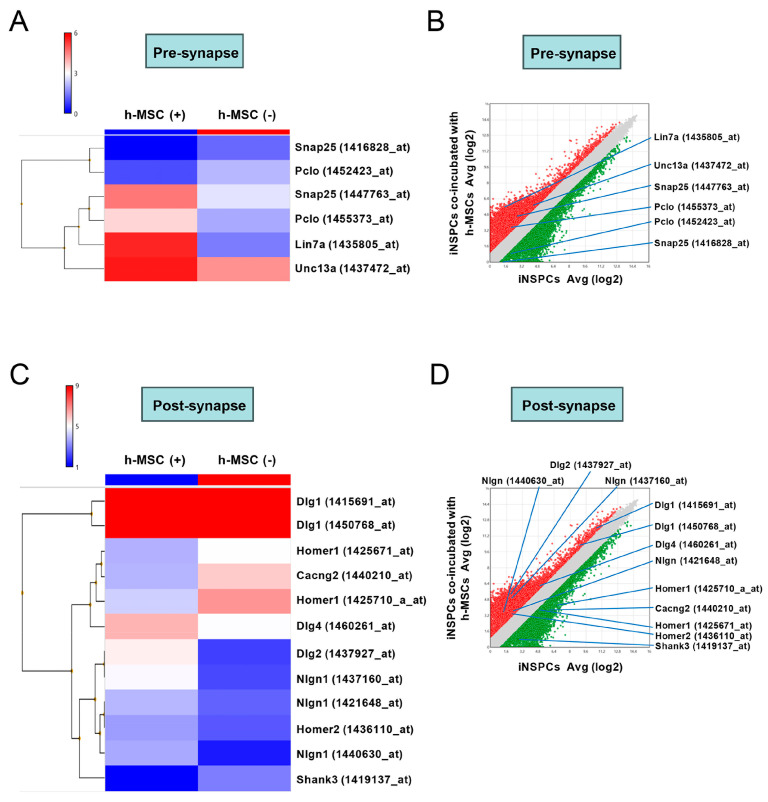
(**A**,**C**) Heat map analysis of the gene expression patterns before (**A**) and after synapse (**C**) in iNSPCs alone and coincubation with h-MSCs. (**B**,**D**) Scatter plot analysis reveals that the genes associated with pre-synapse (**B**) or post-synapse (**D**) are located at the regions >2-fold higher (red plots) or 2-fold lower (green plots) in iNSPCs after coincubation with h-MSCs relative to iNSPCs alone. Abbreviations: iNSPC, injury/ischemia-induced neural stem/progenitor cell; MSC, mesenchymal stem cell.

We further investigated the expression patterns of the synapse-related genes of iNSPCs. The pathway analysis by microarray analysis showed that various pre- and post-synaptic-related genes were upregulated and/or downregulated in iNSPCs after incubation with h-MSCs (Figure 7A). Similarly, heatmapping (Figure 8A,C) and scatter plot analysis (Figure 8B,D) showed that pre- (Figure 8A,B) and post-synaptic-related genes (Figure 8C,D) were significantly upregulated (>2-fold, red dots in Figure 8B,D) and/or downregulated (<−2-fold, green dots in Figure 8B,D) in iNSPCs after incubation with h-MSCs.

Furthermore, GO analysis showed that categories, including “positive regulation of synaptic vesicle transport” [GO term (1902805), p (5.48E-4), enrichment (5.16), genes (*Slc4a8*, *Syt2*, *Nlgn1*, *Syt7*, *Rims1*, *Bcl2l1*)] and “positive regulation of synaptic vesicle exocytosis” [GO term (2000302), p (5.48E-4), enrichment (5.16), genes (e.g., *Slc4a8*, *Syt2*, *Nlgn1*, *Syt7*, *Rims1*, and *Bcl2l1*)] were enriched in iNSPCs cocultured with h-MSCs than in iNSPCs monocultured. These results indicate that h-MSCs regulate the expression of synapse-related genes of iNSPCs, suggesting that various neuronal transmitters are activated in iNSPC-derived neurons by h-MSCs.

## 4. Discussion

Ischemic stroke causes severe brain damage, including neuronal cell death. Although neural regeneration may be limited after ischemic stroke, we previously demonstrated that endogenous iNSPCs are activated within and around ischemic areas and can contribute to neural regeneration after ischemic stroke [17,19]. Although neural regeneration by iNSPCs is usually limited after ischemic stroke, accumulating evidence shows that transplantation of several cell types, including endothelial cells [37] and human brain-derived ischemia-induced stem cells (h-iSCs) [21], improves neurological function by activating iNSPCs. In this study, we demonstrated for the first time that h-MSC transplantation improved neurological function after ischemic stroke, presumably by regulating the fate of iNSPCs.

MSCs are stem cells widely distributed in various organs, such as bone marrow [38], adipose tissue [39], and umbilical cord [40]. MSCs can differentiate into multilineage cells, including neural lineages [41,42]. Several preclinical studies using animal models of central nervous system diseases have shown that MSC transplantation has beneficial effects on injured brains via various mechanisms, including neuroprotective effects [43,44] and neural reparative effects [41,45]. Although the mechanism by which transplanted MSCs exerted neuroprotective effects on post-stroke brains remains unclear, transplanted MSCs decreased neural cell death by secreting trophic factors (e.g., brain-derived neurotrophic factor and glia-derived neurotrophic factor) and chemotactic cytokines [43,46]. Furthermore, transplanted MSCs promoted neural repair by enhancing the proliferation, migration, and differentiation of NSPCs in the SVZ and SGZ [13,14,15].

Besides NSPCs in conventional neurogenic zones, such as the SVZ and SGZ, we previously showed that regionally activated iNSPCs can significantly contribute to neural regeneration after brain injuries, such as ischemic stroke [17,18,19]. However, little is known about whether transplanted MSCs affect the fate of locally activated endogenous iNSPCs after ischemic stroke. In this study, to elucidate this, we transplanted h-MSCs into post-stroke nestin–GFP transgenic mice. We found that MSC transplantation activated endogenous iNSPCs around the grafted sites. Similar to these findings, we recently showed that h-iSC transplantation activated endogenous iNSPCs around the grafted sites but not NSPCs in the SVZ [21]. These findings suggest that although the precise traits of endogenous iNSPCs activated by MSC transplantation remain unclear, not only h-iSCs but also h-MSCs activate locally derived endogenous NSPCs, rather than SVZ-derived NSPCs, after cell transplantation. Although the mechanism by which MSC transplantation activates iNSPC remains unclear, studies have shown that MSCs have immunomodulatory effects via various mechanisms (e.g., secretion of cytokines and chemokines [47] and upregulated and downregulated expression of anti- and proinflammatory cytokines [43,48]). Furthermore, transplanted MSCs exert immunomodulatory effects by promoting the conversion of endogenous MGs/MΦs into the anti-inflammatory M2 type [49]. Because we recently demonstrated that MGs/MΦ (in particular, proinflammatory M1 type) negatively regulated the proliferation of iNSPCs [22], grafted MSCs may suppress the proinflammatory effect of MGs/MΦ, thereby enhancing the activation of endogenous iNSPCs.

Interestingly, in this study, we found that several immune system-related signaling pathways (e.g., “TNF-alpha NF-κB signaling pathway”, “Toll-like receptor signaling pathway”, and “IL-6 signaling pathway”) were significantly upregulated in iNSPCs after co-incubated with h-MSCs. These results suggest that h-MSCs can directly regulate the fate of iNSPCs by activating immune system-related pathways. However, the precise traits of iNSPCs influenced by these signaling pathways should be determined in future studies.

Consistent with in vivo findings, this study displayed that h-MSCs promoted the proliferation of iNSPCs in coculture experiments. A previous study showed that MSCs promoted the proliferation of human-derived NSPCs isolated from the periventricular region by Notch-1 signaling activation [32]. Furthermore, an interaction between the Notch, epidermal growth factor receptor (EGFR) [29], and Hedgehog signaling pathways [33] has been reported to regulate the number and self-renewal of NSPCs in the SVZ and SGZ. In this study, the “EGFR1 signaling pathway”, “Hedgehog signaling pathway”, “Delta-Notch signaling pathway”, and “Notch signaling pathway” were significantly enhanced in iNSPCs after cell–cell interaction with h-MSCs. Thus, MSCs may regulate the number of iNSPCs in a similar manner to NSPCs in the SVZ and SGZ [29,32,33].

In this study, the pathway analysis showed that h-MSCs are involved in the regulation of “integrin-mediated cell adhesion” in iNSPCs. Furthermore, we found that the gene expression of neural cell adhesion molecule 1 (N-cadherin1, *NCAM1*) was significantly upregulated (3.44-fold) in iNSPCs co-incubated with h-MSCs compared with that in iNSPCs alone. Because integrin families, including NCAM, play an important role in the maintenance of NSPCs in the SVZ, integrin signaling pathways may also be associated with the increased number of iNSPCs co-incubated with h-MSCs.

In this study, the coculture experiments showed that h-MSCs promoted the neural differentiation of iNSPCs. Although the precise mechanism remains unclear, studies have shown that the Wnt [34] and Akt signaling pathways [35,36] are related to the neural differentiation of hippocampus-derived NSPCs. Because iNSPCs cocultured with h-MSCs also displayed the activation of these signaling pathways, MSCs may be involved in the regulation of the neural differentiation of iNSPCs, partially in a similar manner.

This study demonstrated that h-MSCs promote the astrocytic differentiation of iNSPCs. In addition, h-MSCs induced the upregulated expression of genes related to astrocytic lineages in iNSPCs. These results are consistent with those of previous studies, which have reported that MSC promoted the differentiation of various types of NSPCs (e.g., hippocampus-derived NSPCs [50] and NSPC cell lines [40]) into glial lineages. Although the mechanism by which h-MSCs enhance the expression of astrocytic markers in iNSPCs after cell–cell interaction remains unclear, STAT3 signaling [51] and FGF8 binding to FGFR3 [52] were reported to be responsible for the acquisition of astrocytic lineages. Although the gene expression level of *STAT3* was not significantly different between iNSPCs cocultured with h-MSCs and iNSPCs alone, the gene expression level of *FGF8* was significantly upregulated (16.52-fold) in iNSPCs cocultured with h-MSCs compared with that in iNSPCs alone. However, MSC-derived effects on iNSPCs regarding specific lineage differentiation should be elucidated in future studies.

In this study, we found that MSC transplantation improved neurological function in post-stroke mice. Although the mechanism remains unclear, the coculture experiments showed that h-MSCs promote the neural differentiation of iNSPCs. In addition, the coculture experiments revealed that h-MSCs regulated the expression patterns of pre- and post-synaptic genes in NSPCs. Furthermore, GO analysis showed that “positive regulation of synaptic vesicle transport” and “positive regulation of synaptic vesicle exocytosis” were enriched in iNSPCs cocultured with h-MSCs compared with those in iNSPCs alone. In this study, as mentioned above, *NCAM1* was significantly upregulated in iNSPCs cocultured with h-MSCs compared with that in iNSPCs alone. Because NCAM plays an important role in synaptic maturation [53,54], MSCs may promote synaptic formation in NSPC-derived neurons in this manner. Thus, neurological function improvement may be partially due to synapse-derived neuronal transmitters that were activated in iNSPC-derived neurons treated with h-MSCs.

This study has several limitations. First, the dose-dependent effect of h-MSCs on iNSPCs was not evaluated. Second, the medium used for the coculture experiments contained 2% FBS, which may be more suitable for iNSPCs than MSCs. Third, we obtained the iNSPC samples for microarray analysis under conditions for proliferation rather than for differentiation. Therefore, the effects of h-MSCs on iNSPCs in coculture experiments may vary with the culture conditions (e.g., cell numbers, serum concentration, conditions for promoting proliferation or differentiation). Furthermore, the precise fate of endogenous iNSPCs activated by transplanted h-MSCs should be elucidated in vivo, ideally by genetic fate-mapping studies using a specific marker for iNSPCs. Alternatively, co-transplantation studies by iNSPCs and h-MSCs into post-stroke animals may be useful in clarifying the roles of h-MSCs against iNSPCs in vivo.

In conclusion, we showed that h-MSC transplantation exhibits positive effects on neurological improvement after ischemic stroke, presumably by regulating the fate of iNSPCs. Although the precise roles of MSCs on endogenous iNSPCs remain unclear, understanding the relationships between MSCs and iNSPCs may provide a novel strategy for improving iNSPC-based neural repair after MSC transplantation in pathological brains, such as ischemic stroke.

## Data Availability

The data supporting this article will be shared by the corresponding author upon reasonable request.
